# P-3. Assessing the Public Health Impact of Transitioning from a 4-valent to a 9-valent HPV Vaccination Program in Panama

**DOI:** 10.1093/ofid/ofae631.214

**Published:** 2025-01-29

**Authors:** Juan Carlos Orengo, Ana Marisol Rendon, Bruna Cristina Lima, Vincent Daniels, Kunal Saxena, Andrew Pavelyev, Cintia I Parellada

**Affiliations:** MSD (IA) LLC, Guaynabo, Puerto Rico; MSD Panama, Panama, Panama, Panama; MSD Brazil, São, Sao Paulo, Brazil; Merck&Co.,Inc., Rahway, New Jersey; Merck&Co.,Inc., Rahway, New Jersey; Merck&Co.,Inc., Rahway, New Jersey; MSD Brazil, São, Sao Paulo, Brazil

## Abstract

**Background:**

In 2008, a human papillomavirus (HPV) vaccine was introduced for 10-year-old girls in the National Immunization Program in Panama, switching to a quadrivalent HPV gender-neutral vaccination (4vHPV GNV). This analysis aimed to estimate the expected public health and economic impact of switching from a 4vHPV GNV to 9vHPV GNV in Panama.

**Table 1.**
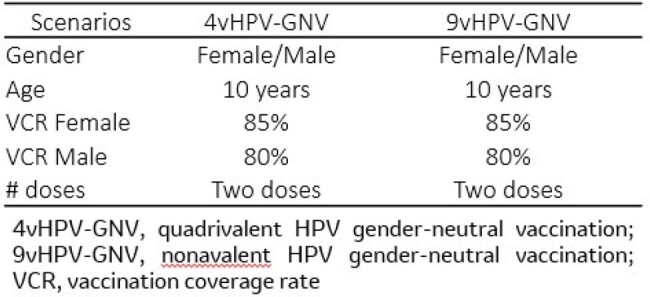
Main parameters used in the model

**Methods:**

A previously published dynamic model of HPV disease transmission was calibrated to simulate the natural history of HPV and attributable disease burden in Panama. The model assumed a two-dose schedule over a 100-year time horizon, lifetime immunity after vaccination, continuous cytology screening, and herd immunity. The outcomes measured were incremental averted cases and deaths of cervical, vaginal, vulvar, anal, penile, head/neck HPV-attributable cancers, recurrent respiratory papillomatosis, genital warts, and cervical intraepithelial neoplasia (CIN) 1 and 2/3. Panama specific data were used for calibration (Table 1).

**Table 2.**
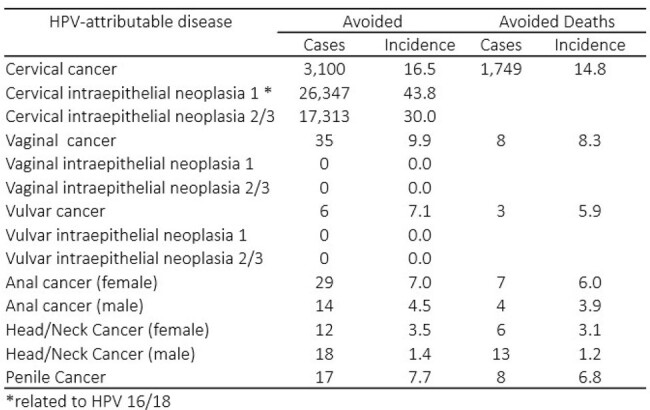
Additional avoided cases/cumulative percent reduction and deaths/mortality percnt reduction of HPV-attributable diseases and cancers with 9v-HPV relative to 4v-HPV strategy over 100 years in Panama

**Results:**

After 100 years, the 9vHPV GNV scenario is estimated to provide faster and greater reductions in the incidence and mortality of HPV 6/11/16/18/31/33/45/52/58-attributable diseases and cancers compared to the 4vHPV GNV. The fastest and greatest cumulative reduction compared to 4vHPV GNV were observed in cervical cancer, CIN 1, and CIN 2/3, totaling 46,760 averted cases and 1,760 deaths (Table 2 and Fig.1). The other cancers also exhibited a reduction in cumulative incidence compared to 4VHPV GNV at year 100, albeit at a later stage than cervical cancer (Fig. 2).

**Figure 1.**
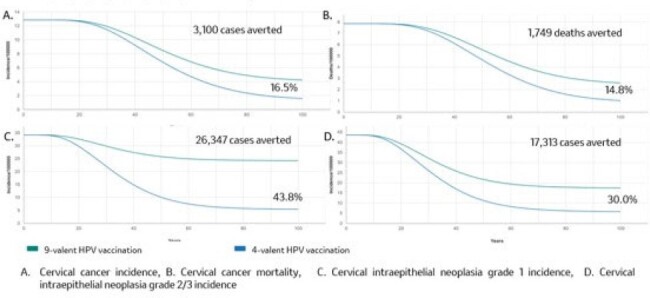
Estimated reductions in incidence and mortality of cervical cancer and cervical intraepthelial neoplasia attributable to HPV 6/11/16/18/31/33/45/52/58 over 100 years in Panama

**Conclusion:**

In Panama, switching from 4vHPV GNV to 9vHPV GNV is projected to provide a substantial public health impact in terms of reduction of HPV-attributable disease and cancers due to HPV 6/11/16/18/31/33/45/52/58 in both genders compared to the current strategy. Faster and greater reductions in the incidence and mortality of cervical cancer and its precursors were seen compared to anal, penile, and head/neck cancers, which typically peak in the sixth decade of life versus fourth decade in cervical cancer, reflecting the natural history of HPV infection in different anatomical sites.

**Figure 2.**
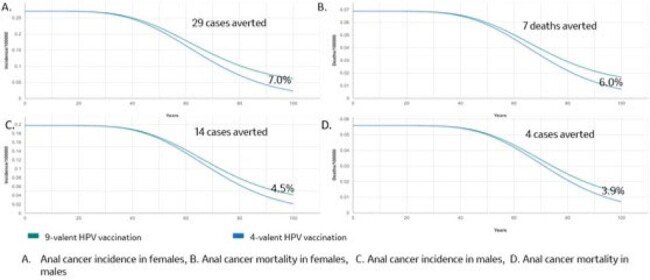
Estimated reductions in incidence and mortality of anal cancer attributable to HPV6/11/16/18/31/33/45/52/58 over 100 years in Paranama

**Disclosures:**

**Juan Carlos Orengo, MD, MPH, PhD**, Merck & Co., Inc: Employee|Merck & Co., Inc: Stocks/Bonds (Private Company) **Ana Marisol Rendon, MD, MS**, Merck & Co., Inc: Employee|Merck & Co., Inc: Stocks/Bonds (Private Company) **Bruna Cristina Lima, n/a**, Merck & Co., Inc: Employee **Vincent Daniels, PhD**, Merck & Co., Inc: Employee|Merck & Co., Inc: Stocks/Bonds (Private Company) **Kunal Saxena, PhD**, Merck & Co., Inc: Stocks/Bonds (Private Company) **Andrew Pavelyev, n/a**, HCL America, Inc.: emplo **Cintia I. Parellada, MD, PhD**, Merck & Co., Inc: Employee|Merck & Co., Inc: Stocks/Bonds (Private Company)

